# Liposome-Based Photodynamic Therapy for Breast Cancer: Innovations in Targeted Delivery, Combination Strategies, and Clinical Translation

**DOI:** 10.3390/ijms27114763

**Published:** 2026-05-25

**Authors:** Nehla Banu, Elder de la Rosa, Muhammad Azeem Saeed, Pedro Salas, Sandeep Surendra Panikar

**Affiliations:** 1 Department of Medicine, Division of Oncology, Washington University of Medical School, 660 S. Euclid Ave., St. Louis, MO 63110-1010, USAsaeed@wustl.edu (M.A.S.); 2 Facultad de Ingenierías y Tecnologías, Universidad La Salle Bajío, León 37150, Guanajuato, Mexico; 3 Centro de Física Aplicada y Tecnología Avanzada, Universidad Nacional Autónoma de México, Apartado Postal 1-1010, Santiago de Querétaro 76000, Querétaro, Mexico

**Keywords:** breast cancer, photodynamic therapy (PDT), photosensitizer, combinational therapy

## Abstract

Breast cancer remains a leading cause of cancer-related mortality worldwide, with treatment resistance, recurrence, and metastasis significantly limiting the effectiveness of conventional therapies. Photodynamic therapy (PDT) has emerged as a minimally invasive and highly selective approach, utilizing photosensitizer-generated reactive oxygen species (ROS) to achieve precise tumor cytotoxicity while preserving surrounding healthy tissue. However, clinical translation of PDT remains constrained by critical biological barriers within the tumor microenvironment, including tumor hypoxia, limited light penetration, poor photosensitizer stability, and inefficient cellular uptake. Antigen-targeted liposomal nanocarriers offer a compelling solution by enabling targeted drug delivery and tumor-specific photosensitizer accumulation, prolonged systemic circulation, and enhanced cellular internalization. Their multifunctional architecture uniquely supports combinational therapeutic strategies, integrating PDT with chemotherapy, photothermal therapy, gene therapy, X-ray-induced photodynamic therapy (X-PDT) and immune checkpoint blockade, thereby amplifying antitumor efficacy and overcoming drug resistance mechanisms. This review comprehensively summarizes recent advances in liposome-based PDT for breast cancer, highlighting multimodal therapeutic integration. Special emphasis is placed on preclinical and emerging clinical outcomes, pilot-scale manufacturing considerations, and strategies to minimize immune clearance.

## 1. Introduction

Nanotechnology has emerged as a powerful interdisciplinary approach offering novel opportunities for the diagnosis and treatment of breast cancer [1,2,3]. In particular, nanostructure-enabled Photodynamic Therapy (PDT) has attracted considerable attention as a minimally invasive treatment modality that combines a photosensitizer, light of an appropriate wavelength, and molecular oxygen to generate cytotoxic reactive oxygen species (ROS) capable of inducing localized tumor cell death [4,5]. The integration of PDT with targeted nanocarrier systems has significantly improved treatment selectivity, therapeutic efficacy, and safety profiles. Liposome-based nanoplatforms have emerged as a promising strategy for photodynamic therapy (PDT)–centered breast cancer theranostics, owing to their compatibility with drug delivery and imaging applications [4]. Among the various nanocarriers investigated, liposomes are among the most clinically advanced and versatile delivery systems for PDT. Their biocompatibility, structural similarity to biological membranes, and tunable size enable efficient encapsulation of photosensitizers within either the aqueous core or lipid bilayer, depending on their physicochemical properties. Importantly, liposomes protect photosensitizers from premature degradation, improve the solubility of hydrophobic photosensitizing agents, and enhance tumor accumulation, thereby addressing key limitations of conventional PDT.

Liposomes are typically composed of natural or synthetic phospholipids combined with cholesterol, providing structural integrity and controlled release behavior [6]. By modifying lipid composition, liposomal formulations can influence absorption, distribution, metabolism, and excretion (ADME) profiles, ultimately improving photosensitizer bioavailability and therapeutic response [7]. However, unmodified liposomes are rapidly cleared from systemic circulation due to opsonin adsorption and uptake by the mononuclear phagocyte system (MPS), limiting their effectiveness for PDT applications that require sufficient tumor accumulation. To overcome these challenges, surface modification with hydrophilic polymers such as polyethylene glycol (PEG) has become a standard strategy. PEGylation significantly reduces protein adsorption and phagocytic recognition, prolongs circulation time, and enhances passive tumor targeting via the enhanced permeability and retention (EPR) effect [8]. PEGylated liposomes have therefore emerged as highly effective carriers for photosensitizers in PDT, enabling increased tumor localization and improved light-activated therapeutic outcomes [9].

Conventional breast cancer treatments, including surgery, radiotherapy, and chemotherapy, remain effective for early-stage disease but are often associated with significant systemic toxicity and limited efficacy in metastatic settings [10,11]. Photodynamic therapy offers several advantages over these modalities, including spatial and temporal control of treatment, reduced systemic side effects, and the potential to stimulate antitumor immune responses [12]. Nevertheless, the clinical success of PDT is hindered by inadequate photosensitizer delivery, poor tumor selectivity, and limited penetration depth of light [13]. Targeted liposomal delivery systems address these limitations by conjugating ligands such as monoclonal antibodies, peptides, aptamers, vitamins, or glycoproteins to the liposomal surface. These ligands selectively recognize biomarkers overexpressed in breast cancer, including human epidermal growth factor receptor 2 (HER2), estrogen receptors (ER), and folate receptors [14]. Human epidermal growth factor receptor 2 (HER2), a member of the ErbB receptor tyrosine kinase family, is overexpressed in approximately 15% of breast cancers and is associated with aggressive tumor behavior, making it an attractive target for photodynamic therapy (PDT)–based nanotherapeutics [15]. Although treatment advances have improved survival outcomes in early-stage HER2-positive breast cancer, long-term follow-up shows that 15–24% of patients still experience disease recurrence [16]. However, long-term prognostic factors in nonmetastatic HER2-positive breast cancer remain poorly defined. This study aimed to assess clinical and pathological determinants of long-term outcomes in this patient population [16].

Liposomes incorporating photosensitizers and targeting ligands enable site-specific photodynamic activation, resulting in enhanced ROS generation and selective tumor ablation upon light irradiation [17,18]. Furthermore, the incorporation of functional nanoparticles such as gold or semiconductor nanoparticles into PDT-active liposomes facilitates multimodal theranostic applications, including fluorescence imaging, photoacoustic imaging, and synergistic photothermal–photodynamic therapy [19].

Overall, in this review, we discuss recent advances in liposome-based immunonanostructures as promising platforms for photodynamic therapy–focused breast cancer theranostics. Particular emphasis is placed on targeted photosensitizer delivery, strategies for enhancing reactive oxygen species generation, immune evasion, and the integration of diagnostic and therapeutic functionalities within a single nanoplatform. By critically analyzing current design approaches and remaining challenges, this review aims to highlight future directions for the development and clinical translation of PDT-centered liposomal nanotherapeutics for breast cancer management.

## 2. Breast Cancer Liposomes

**Figure 1:**
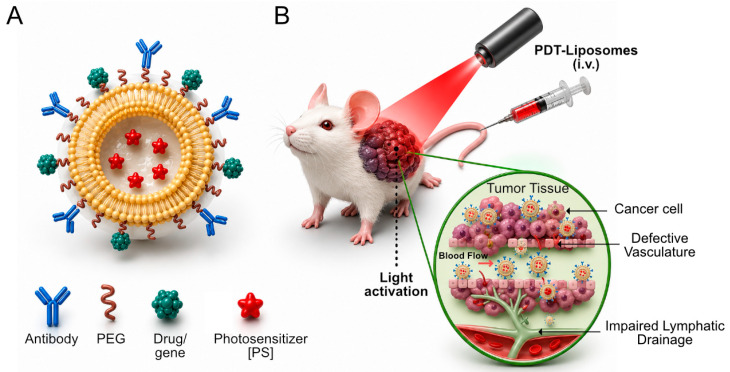
Schematic representation of Targeted liposome nanocarriers for PDT-based breast cancer therapy. (**A**) Structural components of a PDT-targeted liposome, incorporating antibody targeting ligands, PEG coating, encapsulated drug/gene, and photosensitizer. (**B**) In vivo mechanism of intravenously administered PDT-targeted liposomes, illustrating tumor-selective accumulation via the enhanced permeability and retention (EPR) effect through defective tumor vasculature and impaired lymphatic drainage, followed by light activation for localized tumor cytotoxicity.

Liposomal nanostructures represent one of the most extensively investigated and clinically validated nanocarrier systems for cancer therapy, including photodynamic therapy (PDT), Figure 1 [17,18]. Their unique structural organization, composed of phospholipid bilayers enclosing an aqueous core, allows versatile encapsulation of photosensitizers with diverse physicochemical properties Figure 1. The effectiveness of liposomes in PDT is strongly influenced by lipid composition, membrane fluidity, surface chemistry, and overall physicochemical stability, which collectively determine circulation time, tumor accumulation, intracellular uptake, and reactive oxygen species (ROS) generation [20,21].

The membrane-perturbing behavior of liposomes is governed by lipid chain length, degree of saturation, and thermotropic phase transition characteristics of the lipid bilayer [21,22]. These parameters control membrane permeability, rigidity, and interactions with cellular membranes. The amphipathic nature of lipids facilitates fusion or endocytic internalization, enabling intracellular localization of photosensitizers, an essential requirement for efficient PDT, as ROS have a short diffusion distance and limited lifetime [23]. Advances in lipid chemistry have enabled the rational design of liposomes with enhanced stability and reduced premature leakage of photosensitizers during systemic circulation. Saturated phospholipids with chain lengths ranging from C8 to C20, often combined with cholesterol, improve membrane packing and rigidity, thereby preserving photosensitizer integrity [24,25]. Large unilamellar vesicles (LUVs) composed of lipids such as 1,2-dipalmitoyl-sn-glycero-3-phosphocholine (DPPC) have demonstrated favorable delivery characteristics and cytotoxic responses in breast cancer cell models [26], highlighting the importance of lipid selection in PDT-oriented liposomal systems.

Photosensitizers may be encapsulated either within the aqueous core or embedded within the lipid bilayer, depending on their hydrophilicity [17,27]. Liposomal encapsulation improves aqueous solubility of hydrophobic photosensitizers, protects them from photobleaching and enzymatic degradation, and reduces nonspecific phototoxicity in healthy tissues. These advantages address several key limitations of conventional PDT and enhance its therapeutic index.

### 2.1. Liposome-Based Strategies for Breast Cancer

The heterogeneity and complex progression patterns of breast cancer necessitate targeted therapeutic approaches that can selectively eradicate malignant cells while sparing normal tissues. Active targeting strategies exploit molecular biomarkers overexpressed on breast cancer cells to enhance the selective delivery of liposomal photosensitizers. Such approaches are particularly valuable in PDT, where precise localization of the photosensitizer determines the spatial selectivity of light-induced cytotoxicity. Active targeting is achieved by conjugating specific ligands, including monoclonal antibodies [28], antibody fragments [29], VNARs [30], aptamers [31], peptides [17,18], or carbohydrates [32,33,34] to the liposomal surface. Trastuzumab (Herceptin^®^) was among the first FDA-approved therapeutic monoclonal antibodies for the treatment of breast cancer [35,36]. Antibody-based therapy targeting HER2 using recombinant humanized anti-HER2 antibodies has demonstrated strong clinical efficacy, establishing HER2 as a robust biomarker and therapeutic target in breast cancer [37,38]. HER2 is overexpressed in approximately 25–30% of breast cancers and is associated with aggressive tumor behavior, poor prognosis, and reduced overall and disease-free survival. Therapeutic antibodies directed against HER2 have been shown to inhibit tumor xenograft growth and suppress transformed cells that overexpress the HER2 receptor [39]. Clinically, only a limited number of liposomal formulations, such as Doxil^®^ [40], Myocet^®^ [41], and Lipo-Dox^®^ [42], have achieved regulatory approval, while several others, including ThermoDox^®^ [43], EndoTAG-1 [44], MM-302 [45], and DPX-0907 [46] remain in various stages of clinical evaluation. Notably, certain candidates such as TLC-D99 (Evacet^®^) [47] did not progress to approval, highlighting the translational gap between clinical development and successful market authorization. Clinically approved and investigational liposomal formulations used in breast cancer therapy further support this translational relevance, as summarized in Table 1.

The murine monoclonal antibody (mAb) 4D5, which targets the extracellular domain of HER2, effectively inhibited the proliferation of HER2-overexpressing cells [48]. However, the clinical application of murine antibodies is limited due to their immunogenicity. To overcome this limitation, mAb 4D5 was humanized by grafting its complementarity-determining regions (CDRs) into the framework of a consensus human immunoglobulin G, resulting in a recombinant antibody with enhanced HER2-binding affinity compared to the original murine antibody [48]. Furthermore, combination therapy using recombinant humanized anti-HER2 antibodies with chemotherapeutic agents such as paclitaxel and doxorubicin demonstrated significantly enhanced antitumor activity [37].

In addition to HER2-targeted strategies, monoclonal antibody therapies targeting epidermal growth factor receptor (EGFR), which is overexpressed on the surface of several cancer cells, have also been developed [49]. Human IgG2 anti-EGFR monoclonal antibodies, i.e., Panitumumab, exhibit potent neutralizing capabilities and effectively inhibit tumors that depend on EGFR signaling for sustained growth and progression [50]. Notably, improved therapeutic outcomes have been reported in trastuzumab-resistant HER2-positive breast cancer patients when trastuzumab was combined with the small-molecule tyrosine kinase inhibitor lapatinib [51]. Triple-negative breast cancer (TNBC) is a clinically challenging subtype defined by the absence of estrogen receptor, progesterone receptor, and HER2 expression, which greatly limits the availability of targeted treatment options [52,53]. It is often characterized by aggressive tumor behavior, a higher risk of metastasis, and a highly heterogeneous and immunosuppressive tumor microenvironment [54,55]. Together, these features strongly influence the delivery and therapeutic performance of photodynamic therapy and nanocarrier-based systems [56,57]. In particular, conditions such as hypoxia, dense stromal architecture, and abnormal tumor vasculature can restrict photosensitizer accumulation and light penetration, ultimately reducing treatment efficacy [58]. For this reason, active targeting strategies and rational nanocarrier design are especially important for improving therapeutic selectivity and overcoming microenvironment-driven resistance in TNBC [59].

Despite these advances, drug resistance remains a major challenge in breast cancer therapy. Resistant cancer cells often overexpress P-glycoprotein, a membrane-associated efflux transporter encoded by the MDR1 gene, which actively pumps chemotherapeutic agents out of cells, thereby reducing intracellular drug accumulation and efficacy [60,61]. To address this issue, liposomal delivery systems have been developed to simultaneously silence genes responsible for multidrug resistance [62,63,64,65] and deliver chemotherapeutic agents after sensitizing cancer cells [66]. Such combinational nanotherapeutic strategies represent a promising approach for downregulating chemoresistance mechanisms while enhancing targeted drug delivery.

Specifically, liposomal nanostructures have been designed to target doxorubicin-resistant cancer cells by recognizing Specifically, liposomal nanostructures have been designed to target doxorubicin-resistant cancer cells by recognizing the extradomain B (EDB) of fibronectin, which is overexpressed in chemoresistant tumors [67]. These systems enable the co-delivery of doxorubicin and siRNA targeting MDR1, effectively silencing P-glycoprotein expression and resensitizing cancer cells to chemotherapy [68]. Evaluation of these liposomal formulations demonstrated superior targeting efficiency toward the EDB domain in chemoresistant cancer cells, highlighting their potential for overcoming multidrug resistance [69]. Building on the clinical success of antibody-based targeting strategies, increasing attention has shifted toward alternative ligands for nanocarrier functionalization. Among these ligands, small peptides have gained increasing interest due to their improved stability, lower immunogenicity, and ease of chemical modification compared with full-length antibodies.

**Table 1:** Overview of Liposomal Formulations in Breast Cancer Therapy: Regulatory Approval Status and Clinical Development.

Compound/Drug	Commercial Name	Carrier	Clinical Trial Status	Brand/Manufacturer	Reference
Annamycin	Annamycin	Liposomes	Phase I/II	Aronex, USA	[70]
Doxorubicin	TLC-D99 (Evacet^®^)	Liposomes	FDA filed	Elan Corporation, Ireland	[47]
Doxorubicin	Doxil^®^/Caelyx^®^	Liposomes	Approved (Canada & Europe, 2003)	ALZA, Schering-Plow, USA	[40,41]
Doxorubicin + Cyclophosphamide	Myocet^®^	Liposomes	Approved (Europe, 2003)	Elan, Ireland	[71]
Doxorubicin	Lipo-Dox^®^	Liposomes	Approved (Taiwan, 2001)	–	[42]
Thermosensitive Doxorubicin	ThermoDox^®^	Thermosensitive Liposomes	Phase III	Celsion Corporation, USA	[43]
Paclitaxel	EndoTAG-1	Cationic Liposomes	Phase II	Medigene, Germany	[44]
Doxorubicin	2B3-101	Liposomes	Phase I/II	–	[72]
Doxorubicin	MM-302	Liposomes	Phase I	Merrimack Pharmaceuticals, USA	[45]
HLA-A2-restricted peptides + T-helper peptide + polynucleotide adjuvant	DPX-0907	Liposomes (Vaccine)	Phase I	–	[46]

Ligand-mediated endocytosis of liposomes significantly increases intracellular photosensitizer concentration, leading to enhanced ROS production upon light irradiation and improved PDT efficacy. In addition, targeted liposomes can reduce off-target photosensitizer accumulation, thereby minimizing damage to surrounding healthy tissues. The integration of active targeting with surface modifications such as polyethylene glycol (PEG) further optimizes liposomal performance. PEGylation reduces nonspecific protein adsorption and immune recognition while maintaining ligand accessibility, enabling prolonged circulation and enhanced tumor localization.

### 2.2. Photosensitizers for Breast Cancer Photodynamic Therapy

The choice of photosensitizer is a critical determinant of PDT efficacy [73]. Ideal photosensitizers should exhibit strong absorption in the therapeutic window (600–800 nm), high quantum yield for singlet oxygen generation, minimal dark toxicity, and preferential accumulation in tumor tissue. However, many clinically relevant photosensitizers suffer from poor aqueous solubility, aggregation, and nonspecific biodistribution, limiting their therapeutic potential.

Liposomal encapsulation has been widely employed to overcome these limitations. First-generation photosensitizers, such as porphyrin derivatives, and second-generation agents, including chlorins, bacteriochlorins, and phthalocyanines, have been successfully incorporated into liposomal systems [73]. Encapsulation enhances solubility, stabilizes the photosensitizer against degradation, and improves tumor-selective accumulation [73]. More recently, third-generation photosensitizers have been developed by combining photosensitizing agents with targeting ligands or nanocarriers to further enhance selectivity and therapeutic efficacy. Liposome-based delivery of these advanced photosensitizers enables precise control over intracellular localization and facilitates combination strategies involving PDT with chemotherapy, photothermal therapy, or immunotherapy. In this review, we discuss the evolution of photosensitizers for breast cancer PDT and emphasize the role of liposomal nanocarriers in addressing their pharmacokinetic and delivery challenges. A recent study by Jie Yu et al. developed a novel liposomal nanodrug (ICG@ES-Cu Lip) combining photodynamic therapy (PDT) and cuproptosis induction for the treatment of Breast Cancer [74]. The liposomes co-delivered indocyanine green (ICG), a photosensitizer, and elesclomol-copper (ES-Cu), a cuproptosis inducer. Under near-infrared (NIR) irradiation, PDT generated reactive oxygen species (ROS), which significantly enhanced ES-Cu-induced oxidative stress and mitochondrial damage in breast cancer cells [74]. In both cell culture and mouse models, the combined treatment showed stronger tumor-killing activity than either therapy alone, while maintaining good biosafety. The study demonstrated that PDT-enhanced cuproptosis represents a promising synergistic therapeutic strategy for breast cancer [74]. The integration of optimized photosensitizers with targeted liposomal platforms is expected to play a central role in the future development of effective and clinically translatable PDT-based therapies for breast cancer.

## 3. Mechanisms of Photodynamic Therapy in Breast Cancer

Photodynamic therapy (PDT) is a light-activated therapeutic modality that induces localized cytotoxicity through the generation of reactive oxygen species (ROS) [5,73,75]. The therapeutic outcome of PDT is governed by the complex interplay between the photosensitizer, light source, molecular oxygen, and the biological characteristics of the tumor microenvironment [75,76]. In breast cancer, PDT exerts its antitumor effects through multiple interconnected mechanisms, including direct tumor cell killing, vascular damage, and activation of antitumor immune responses. Upon administration, the photosensitizer accumulates within tumor tissue, facilitated by passive or active targeting strategies such as liposome-based delivery systems. Subsequent irradiation with light of an appropriate wavelength excites the photosensitizer from its ground state to an excited singlet state, followed by intersystem crossing to a long-lived triplet state. This excited state initiates photochemical reactions that ultimately result in ROS production, forming the molecular basis of PDT-induced cytotoxicity.

### 3.1. Reactive Oxygen Species Generation and Photochemical Pathways

**Figure 2:**
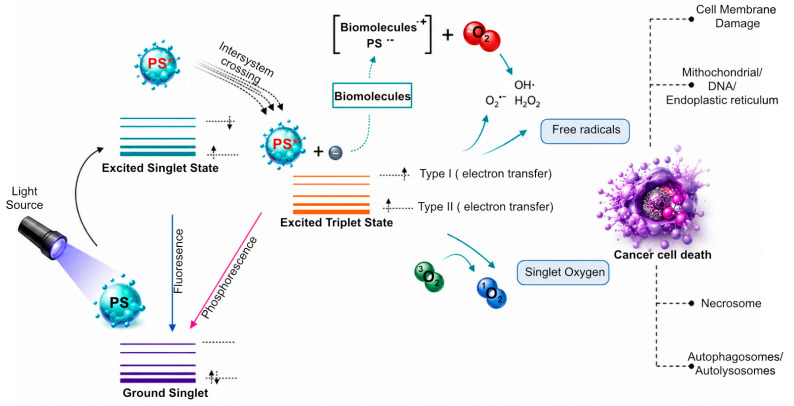
Mechanistic overview of photodynamic therapy (PDT)-induced cancer cell death. Upon light activation, the photosensitizer (PS) is excited from the ground singlet state to the excited singlet state, followed by intersystem crossing to the excited triplet state. This triggers two cytotoxic pathways: Type I reactions involving electron transfer to biomolecules generating free radicals (O^2−^, OH•, H_2_O_2_), and Type II reactions involving energy transfer to molecular oxygen, producing singlet oxygen (^1^O_2_). The resulting reactive oxygen species collectively induce cancer cell death through cell membrane damage, mitochondrial/DNA/endoplasmic reticulum disruption, necrosome activation, and autophagosome/autolysosome formation.

The photochemical processes underlying PDT are generally classified into two primary mechanisms: Type I and Type II pathways, see Figure 2 [77]. In the Type I mechanism, the excited triplet-state photosensitizer undergoes electron or hydrogen transfer reactions with surrounding biomolecules, leading to the formation of free radicals and radical ions, see Figure 2. These reactive intermediates subsequently react with molecular oxygen to generate ROS such as superoxide anions, hydroxyl radicals, and hydrogen peroxide [75,77]. In contrast, the Type II mechanism involves direct energy transfer from the excited triplet-state photosensitizer to ground-state molecular oxygen, resulting in the formation of singlet oxygen (^1^O_2_), Figure 2 [75,77]. Singlet oxygen is considered the predominant cytotoxic agent in most PDT applications due to its high reactivity toward cellular components, including lipids, proteins, and nucleic acids. However, its extremely short lifetime and limited diffusion radius restrict its action to the immediate vicinity of the photosensitizer localization.

The relative contribution of Type I and Type II mechanisms depends on several factors, including the chemical structure of the photosensitizer, oxygen availability, subcellular localization, and local redox conditions [75,77]. Liposome-based delivery systems play a critical role in modulating these parameters by controlling photosensitizer distribution, protecting against aggregation, and enhancing intracellular delivery, thereby optimizing ROS generation efficiency in breast cancer cells.

### 3.2. PDT-Induced Cell Death Pathways

Reactive oxygen species (ROS) generated during photodynamic therapy (PDT) act as the primary cytotoxic mediators, inflicting oxidative damage on vital cellular structures and thereby activating multiple cell death programs [75,77]. Among these, apoptosis is the most commonly observed outcome and is typically initiated through mitochondrial dysfunction, characterized by loss of mitochondrial membrane potential, cytochrome *c* release, caspase cascade activation, and subsequent DNA fragmentation, as shown in Figure 2. Photosensitizers that preferentially localize to mitochondria are therefore particularly potent in engaging apoptotic signaling pathways.

Under conditions of intense photodynamic stress, such as high light fluence or elevated photosensitizer concentrations, PDT can also drive cells toward necrotic death [78]. Necrosis is marked by rapid plasma membrane disruption [12], cellular swelling, and uncontrolled release of intracellular components, which may amplify local inflammatory responses [77,79]. In parallel, PDT has been shown to modulate autophagic processes, which can serve either as a transient cytoprotective response to oxidative injury or, when excessively activated, as an alternative mode of cell death through the formation of autophagosomes and their subsequent fusion with lysosomes [80,81].

Importantly, the dominant cell death pathway elicited by PDT is strongly dictated by the subcellular localization of the photosensitizer. Accumulation at the plasma membrane favors necrotic outcomes, whereas localization within mitochondria [82], lysosomes [83,84], the endoplasmic reticulum [85], or even the nucleus predominantly activates apoptotic signaling [86,87]. Beyond these canonical mechanisms, emerging evidence indicates that PDT can also induce regulated necrosis, or necroptosis, through the assembly of necrosomes containing receptor-interacting protein kinases RIP1 and RIP3. Advances in nanocarrier-based delivery systems, particularly liposomal formulations, provide a powerful strategy to control photosensitizer biodistribution at the subcellular level. By directing photosensitizers to specific organelles, liposomal PDT enables deliberate modulation of cell death pathways, offering a means to optimize therapeutic efficacy while shaping downstream biological responses.

### 3.3. Vascular Effects and Tumor Microenvironment Modulation

Beyond direct tumor cell killing, PDT exerts profound effects on the tumor vasculature. Damage to endothelial cells following PDT leads to increased vascular permeability, vasoconstriction, platelet aggregation, and ultimately vascular shutdown [88]. These effects result in impaired blood flow, nutrient deprivation, and secondary tumor cell death, thereby amplifying the overall therapeutic response. However, PDT-induced vascular damage can also exacerbate tumor hypoxia, which may limit ROS generation due to oxygen depletion. Hypoxia represents a major challenge for PDT efficacy, particularly in poorly perfused breast tumors [89]. Liposome-based delivery strategies and oxygen-generating or oxygen-carrying nanoplatforms have been explored to mitigate hypoxia-associated limitations and enhance PDT performance [90].

### 3.4. Immunogenic Cell Death and Antitumor Immune Responses

An increasingly recognized mechanism of PDT is its ability to induce immunogenic cell death (ICD), thereby stimulating systemic antitumor immune responses [91]. PDT-treated tumor cells release damage-associated molecular patterns (DAMPs), such as calreticulin, heat shock proteins, and high-mobility group box 1 (HMGB1), which promote dendritic cell maturation and antigen presentation [91].

The activation of innate and adaptive immune responses following PDT can lead to long-term tumor control and protection against metastasis [92]. In breast cancer models, PDT has been shown to enhance infiltration of cytotoxic T lymphocytes and natural killer cells into the tumor microenvironment [92]. Liposome-based PDT systems that co-deliver immunomodulatory agents or target immune-related pathways represent a promising strategy for amplifying PDT-induced antitumor immunity [90].

In this review, we highlight the multifaceted mechanisms through which photodynamic therapy exerts therapeutic effects in breast cancer, emphasizing the role of liposomal nanocarriers in enhancing ROS generation, modulating cell death pathways, overcoming microenvironmental barriers, and stimulating immune responses. A comprehensive understanding of these mechanisms is essential for the rational design of next-generation PDT-centered nanotherapeutic strategies.

## 4. Liposomal Drug Delivery Mechanism and Stability

Liposomal formulations represent one of the most extensively studied nanocarrier platforms for targeted drug delivery in cancer therapy, owing to their biocompatibility, versatility in encapsulating hydrophilic and lipophilic molecules, and potential for reduced systemic toxicity [22]. The design of effective liposomes requires meticulous consideration of their structural architecture, lipid composition, surface chemistry, and in vivo disposition, as these factors collectively determine their stability, circulation time, biodistribution, and interaction with the immune system [6,90,93]. One of the primary challenges in liposomal-based PDT delivery is instability during systemic circulation, leading to premature drug release, rapid recognition and clearance by the reticuloendothelial system (RES), and aggregation in physiological environments [94]. In the context of photodynamic therapy (PDT), such instability directly reduces photosensitizer availability at the tumor site, thereby limiting reactive oxygen species (ROS) generation and therapeutic efficacy.

Liposome surface charge, determined by the composition of the lipid head groups and the ambient pH, plays a pivotal role in their in vivo fate [95]. Neutral liposomes exhibit minimal recognition by the RES and lower aggregation tendencies, promoting prolonged systemic circulation. In contrast, negatively charged liposomes, typically composed of phosphatidylglycerol (PG) or phosphatidylserine (PS), are readily recognized by macrophages and other phagocytic cells due to interactions with specific immune receptors, leading to faster clearance [96]. Positively charged lipids, while facilitating interactions with negatively charged cellular membranes, tend to bind serum proteins, resulting in opsonization and rapid RES-mediated clearance [95]. To circumvent immune recognition, liposomes can be modified with glycolipids such as phosphatidylinositol (PI) or ganglioside GM, which inhibit macrophage phagocytosis and prolong circulation in vivo [97]. For PDT applications, prolonged circulation is particularly critical as it increases tumor accumulation of photosensitizers, thereby enhancing light-triggered ROS production.

Polyethylene glycol (PEG) functionalization, or PEGylation, is a widely adopted strategy to enhance liposomal stability, reduce opsonization, and provide a steric barrier against RES-mediated clearance [42]. PEGylation also enables facile conjugation of targeting moieties such as peptides, antibodies, or proteins, facilitating receptor-mediated tumor-specific delivery. In PDT systems, PEGylation further improves intratumoral retention of photosensitizers, thereby increasing spatially confined ROS generation upon irradiation. Optimal PEGylation typically involves PEG chains of molecular weight 1–2 kDa incorporated at 5–10 mol% of total lipid content, creating a hydration layer of approximately 5 nm that shields the liposome surface [98]. This configuration allows effective encapsulation of both hydrophilic and hydrophobic drugs while maintaining nanocarrier stability in the circulation.

The fluidity and phase behavior of liposomal membranes, dictated by the lipid transition temperature (T_m_), are critical determinants of drug release kinetics and membrane integrity [22,99]. Lipid bilayers exhibit a disordered, fluid-like phase above T_m_ and a rigid, gel-like phase below T_m_ [99]. Incorporation of cholesterol modulates membrane fluidity and permeability, stabilizing liposomes and reducing premature drug leakage; however, excessive cholesterol (>30 mol%) can eliminate phase transition, potentially affecting [100] controlled drug release [22]. Liposomes can also be engineered for stimulus-responsive drug release by leveraging T_m_ properties in combination with external triggers such as localized heating (via microwave, infrared, or laser irradiation) to induce phase transitions and facilitate drug unloading at the tumor site [22]. This is particularly relevant for PDT, where light-triggered activation can be coupled with controlled drug release to synchronize photosensitizer availability with irradiation timing, maximizing ROS generation efficiency.

Size is another critical parameter influencing liposomal circulation, biodistribution, and immune recognition. Liposomes with diameters between 50 and 100 nm are generally optimal for evading RES clearance while achieving efficient tumor accumulation through enhanced permeability and retention (EPR) effect. PEGylation can increase the hydrodynamic diameter to 150–200 nm, extending systemic circulation, although eventual clearance by the liver and spleen remains inevitable [8]. For PDT, this size window is particularly important as it balances deep tumor penetration with sufficient photosensitizer payload delivery.

### 4.1. Crossing Immune Barriers and Tumor Microenvironment

Successful delivery of liposomal nanocarriers to tumor cells requires overcoming multiple physiological and cellular barriers within the tumor microenvironment (TME) [101]. In PDT-based systems, these barriers are particularly critical because insufficient tumor accumulation or cellular uptake directly limits intracellular photosensitizer concentration, thereby reducing ROS generation upon light irradiation. Liposomes must evade immune surveillance, penetrate the extracellular matrix (ECM), and undergo cellular internalization to deliver therapeutic payloads effectively. The internalization of liposomes depends on their physicochemical characteristics, such as size, surface charge, and ligand functionalization and the nature of target cells, dictating the predominant uptake pathway, which can be broadly classified into phagocytic and non-phagocytic (endocytic) mechanisms. Efficient internalization ensures site-specific drug release, enhancing cytotoxicity and inhibiting cancer cell proliferation. In PDT, efficient internalization is essential to ensure intracellular photosensitizer localization, which directly determines ROS-induced subcellular damage and cell death pathways.

### 4.2. Phagocytosis and Opsonization

**Figure 3:**
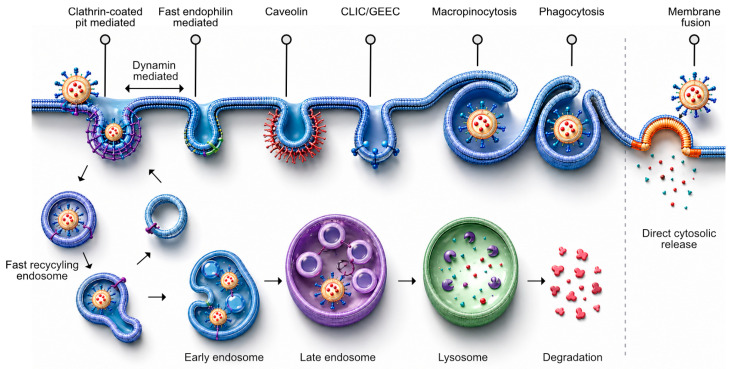
Intracellular uptake pathways of targeted liposome nanocarriers and endosomal escape mechanisms. Immunoliposomes enter cancer cells via multiple endocytic routes, including clathrin-coated pit-mediated (dynamin-dependent) endocytosis, fast endophilin-mediated endocytosis, caveolin-mediated uptake, CLIC/GEEC pathway, macropinocytosis, and phagocytosis. Following internalization, nanocarriers traffic through early endosomes and may undergo fast recycling or progress to late endosomes and lysosomes, where enzymatic degradation risks photosensitizer inactivation. Alternatively, membrane fusion enables direct cytosolic release of the therapeutic payload, bypassing lysosomal degradation and ensuring effective intracellular drug delivery.

Phagocytosis is primarily mediated by professional phagocytes, including macrophages, dendritic cells, monocytes, and neutrophils, though non-professional phagocytes (e.g., fibroblasts, epithelial cells) may also internalize liposomes to a lesser extent, as shown in Figure 3 [102]. For PDT nanocarriers, rapid phagocytic clearance reduces tumor-selective photosensitizer delivery, but can also be exploited for immune activation following PDT-induced immunogenic cell death. Opsonization is a crucial precursor to phagocytosis, wherein serum proteins (opsonins) such as immunoglobulin G (IgG) or complement fragments (C3, C4) tag liposomes for recognition by Fc receptors (FcγR) and complement receptors (CR) on phagocytes [103]. Ligand-receptor interactions activate downstream signaling cascades mediated by Rho-family GTPases, triggering actin polymerization and the formation of pseudopodia that engulf the nanostructure [104]. Following internalization, the actin cytoskeleton depolymerizes from the phagosomal membrane, allowing sequential fusion with early and late endosomes, and ultimately lysosomes, forming phagolysosomes [105]. Within PDT applications, lysosomal processing can influence photosensitizer degradation or activation, thereby affecting intracellular ROS generation efficiency. The acidic environment and enzymatic content within phagolysosomes facilitate payload release and subsequent intracellular activity [106,107].

### 4.3. Non-Phagocytic Endocytic Pathways

Smaller liposomal nanostructures can be internalized via non-phagocytic pathways, including clathrin-mediated endocytosis (CME), caveolae-mediated endocytosis, micropinocytosis, and other clathrin- and caveolae-independent routes, Figure 3 [108]. These pathways are particularly relevant for PDT, as they determine intracellular trafficking routes that control photosensitizer localization to mitochondria, lysosomes, or endoplasmic reticulum, which in turn dictates cell death mechanisms. CME is a receptor-dependent mechanism involving the internalization of ligand-receptor complexes within clathrin-coated pits (~150 nm) [4,109]. Clathrin triskelions assemble into a polyhedral lattice on the cytosolic membrane surface, deforming the membrane into pits, which are then pinched off by dynamin-mediated scission to form clathrin-coated vesicles [110]. These vesicles traffic to acidified early endosomes (pH ~6), mature into late endosomes (pH ~5), and subsequently fuse with prelysosomal compartments containing hydrolases, potentially affecting the stability of sensitive payloads [111]. In PDT, endosomal acidification can either quench or activate photosensitizers depending on their chemical nature, thereby influencing ROS production efficiency. Common targeting ligands exploiting CME include transferrin, epidermal growth factor (EGF), low-density lipoprotein (LDL), and folic acid, particularly in breast cancer cells overexpressing these receptors [112]. Such receptor-mediated uptake is widely exploited in breast cancer PDT to enhance selective intracellular delivery of photosensitizers.

Receptor-independent absorptive pinocytosis, also termed fluid-phase endocytosis, internalizes extracellular fluid and solutes through nonspecific interactions, often involving clathrin-coated pits [113]. Although slower than receptor-mediated CME, this pathway contributes to nanocarrier uptake, particularly for unmodified or neutral liposomes [114].

### 4.4. Caveolae-Mediated Endocytosis

Caveolae-mediated endocytosis serves as an alternative pathway that is particularly suitable for biomolecule-sensitive payloads, such as proteins, peptides, or nucleic acids, Figure 3 [115]. Caveolae are flask-shaped, cholesterol- and sphingolipid-rich invaginations (50–100 nm) on the plasma membrane [28,116,117]. Cargo is internalized through receptor-ligand interactions, and internalized vesicles are released into the cytosol via dynamin-dependent fission, largely avoiding lysosomal degradation [118]. This mechanism is highly advantageous for liposome-based PDT nanocarriers, as it preserves photosensitizer integrity and promotes cytosolic or organelle-specific ROS generation [119,120].

### 4.5. Micropinocytosis

Micropinocytosis is a clathrin- and caveolae-independent, actin-driven endocytic (Figure 3) process commonly utilized by immune cells such as macrophages [113,121]. It involves membrane ruffling and the formation of large vesicles (~1 µm), which can subsequently fuse with lysosomes or recycle to the plasma membrane [122,123]. Micropinocytosis is non-selective but contributes to the internalization of drug-loaded nanocarriers within the tumor microenvironment, enhancing intracellular delivery in regions where receptor-mediated uptake may be limited [122,124]. In aggressive breast cancer subtypes, including TNBC, macropinocytosis can contribute to enhanced nanoparticle uptake, thereby improving PDT responsiveness in otherwise poorly targeted tumor regions.

## 5. Tumor Microenvironment and Biological Barriers

Photodynamic therapy (PDT) has emerged as a promising modality for breast cancer management, offering localized cytotoxicity through the generation of reactive oxygen species (ROS) upon photosensitizer (PS) activation by light [17,18]. However, the clinical efficacy of PDT is often hampered by the complex and heterogeneous tumor microenvironment (TME) and multiple biological barriers that interfere with PS delivery, activation, and intracellular distribution. These barriers, including hypoxia, limited light penetration, impaired cellular uptake, and endosomal entrapment, synergistically reduce ROS production and, consequently, therapeutic outcomes [125]. Understanding the mechanistic interplay between these barriers and PDT efficacy is critical, particularly when designing advanced nanostructures such as liposomes, which offer opportunities for targeted delivery, controlled release, and improved intracellular bioavailability [126]. In this section, we discuss these major challenges in breast cancer PDT and highlight strategies to overcome them using multimodal liposome-based nanocarriers.

### 5.1. Hypoxia

Hypoxia, defined as reduced oxygen availability, is a hallmark of solid breast tumors, arising from abnormal vascular architecture, rapid proliferation, and high oxygen consumption [127]. Since PDT fundamentally relies on the presence of molecular oxygen to generate ROS, hypoxic tumor regions exhibit markedly diminished ROS production, resulting in suboptimal induction of apoptosis and necrosis [128]. Additionally, hypoxia activates hypoxia-inducible factors (HIFs), which promote angiogenesis, metabolic adaptation, and survival signaling pathways, further attenuating therapeutic efficacy [129]. To circumvent these limitations, various oxygen-enhancing strategies have been developed and integrated with nanocarrier systems. Oxygen-carrying platforms, including hemoglobin- or perfluorocarbon-loaded nanoparticles, can deliver molecular oxygen directly to hypoxic tumor regions [130]. In situ oxygen-generating nanoparticles, such as catalase- or MnO_2_-containing constructs, utilize endogenous hydrogen peroxide to produce oxygen locally. Furthermore, hypoxia-activated pro-photosensitizers selectively generate ROS in oxygen-deficient environments, ensuring that PDT cytotoxicity is retained even in the most hypoxic tumor zones [131]. The incorporation of these strategies within liposome-based platforms not only enhances oxygen availability but also allows targeted delivery of PSs, minimizing off-target effects and maximizing therapeutic potential. Recently, Wanwan et al. demonstrated that mitochondrial targeting is indispensable for the anticancer efficacy of TPP-conjugated 2-pyridone endoperoxides, a finding established through systematic variation in the alkyl linker length connecting the singlet oxygen-releasing endoperoxide to the triphenylphosphonium (TPP) unit [132]. Importantly, singlet oxygen is generated via spontaneous thermal cycloreversion rather than by photosensitization, making this approach entirely independent of molecular oxygen availability and therefore effective under the hypoxic conditions that typically limit conventional photodynamic therapy. While all four compounds released singlet oxygen with comparable half-lives (~10 h at 37 °C), only the longer-chain derivatives (Endo-py-tpp-3 and -4) achieved effective mitochondrial localization, as confirmed by Si-DMA fluorescence imaging and JC-1 membrane potential assays, translating into IC_50_ values of 30–60 μM versus >200 μM for the shorter analogs [132]. Apoptosis was mechanistically linked to Bcl-2 downregulation and caspase-3 activation, and activity was retained in 3D tumor spheroids. In a 4T1 murine tumor model, Endo-py-tpp-4 reduced tumor mass by ~75% without significant systemic toxicity, whereas the short-chain Endo-py-tpp-1 was inactive, providing direct in vivo proof that linker-length-dependent mitochondrial access governs therapeutic outcome. However, the study does not explicitly test performance under hypoxic conditions, and the advantage over conventional PDT in low-oxygen tumor microenvironments, while theoretically compelling, remains to be directly validated.

### 5.2. Light Penetration Limitations

The therapeutic efficacy of PDT is inherently dependent on light reaching sufficient depths within tumor tissue to activate photosensitizers. In breast cancer, tissue optical properties, including scattering and absorption by hemoglobin, melanin, and water, restrict light penetration, particularly for conventional visible-light-activated PSs, limiting their application in deeper or locally advanced tumors [133]. Near-infrared (NIR) light offers deeper tissue penetration due to lower absorption and scattering, but uniform activation of PSs throughout the tumor mass remains challenging. To overcome these limitations, advanced strategies such as interstitial light delivery via optical fibers, two-photon excitation, and upconversion nanoparticles that are capable of converting NIR light to shorter wavelengths have been explored [17,134,135]. When combined with liposome-based PS delivery, these strategies enhance PS accumulation specifically within cancer cells and ensure more uniform ROS generation, ultimately improving PDT outcomes [18]. Optimization of light wavelength, intensity, and exposure time is crucial to maximize penetration depth while minimizing damage to surrounding healthy tissues.

### 5.3. Cellular Uptake Barriers

Even when PSs reach the tumor interstitium, their therapeutic efficacy depends on efficient cellular internalization. Breast tumor tissue presents multiple barriers to uptake, including a dense extracellular matrix (ECM) [136], elevated interstitial fluid pressure, and heterogeneous vascular perfusion, which collectively impede diffusion and distribution of PSs [137]. Additionally, the physicochemical properties of PSs, such as hydrophobicity, molecular weight, and surface charge strongly influence their ability to cross cellular membranes. Liposome-based nanocarriers address these challenges by providing a lipid bilayer that enhances PS solubility and stability, while surface functionalization with tumor-specific antibodies or ligands promotes receptor-mediated endocytosis and selective uptake by breast cancer cells. Stimuli-responsive liposomes, designed to release PSs in response to pH changes, enzymatic activity, or redox gradients within the TME, further improve intracellular delivery and minimize off-target effects [138]. Enhanced cellular uptake ensures higher intracellular PS concentrations, which are critical for sufficient ROS generation and effective PDT-mediated cytotoxicity.

### 5.4. Endosomal Escape

Following endocytic uptake, PSs are often sequestered within endosomes or lysosomes, limiting their access to vital organelles such as mitochondria and nuclei, and thereby restricting ROS-mediated cytotoxic effects [139]. Endosomal entrapment represents a major intracellular barrier to effective PDT. To overcome this, several strategies have been developed. Proton sponge-containing polymeric coatings induce osmotic swelling and endosomal rupture, allowing PS release into the cytosol [140]. Fusogenic lipids or peptides destabilize endosomal membranes in response to acidic pH, promoting vesicle disruption [141]. Additionally, photochemical internalization leverages ROS generation at the endosomal membrane upon light activation to facilitate cytosolic release [142]. Liposome platforms can be engineered to incorporate these endosomal escape mechanisms, ensuring that PSs reach the cytosol and organelles critical for apoptosis induction. Improved endosomal escape enhances subcellular ROS delivery, amplifying PDT-induced cytotoxicity and ensuring more consistent therapeutic outcomes across heterogeneous tumor regions.

## 6. Combination Strategies in Breast Cancer Therapy

The therapeutic efficacy of photodynamic therapy (PDT) in breast cancer can be substantially enhanced through combinational approaches with chemotherapy, photothermal therapy (PTT), immunotherapy, and gene therapy [143]. Liposomal and immunoliposomal nanocarriers are particularly suited for such strategies, as they allow co-encapsulation of multiple therapeutic agents, protect sensitive payloads, prolong systemic circulation, and provide tumor-specific delivery [144]. By integrating multiple modalities, these combinational strategies address the inherent limitations of PDT such as hypoxia, limited light penetration, and suboptimal photosensitizer uptake while offering opportunities for theranostic applications [145]. The representative liposomal nanoplatforms employing such combinational strategies in breast cancer are summarized in Table 2. Our group’s work has centered on designing multifunctional nanohybrids that consolidate diagnostic and therapeutic capabilities including NIR-triggered photothermal therapy, nano-thermometry, drug delivery and optical cell tracking into streamlined platforms for precision breast cancer theranostics [17,18,134,135,146].

### 6.1. PDT Combined with Chemotherapy

Combining PDT with chemotherapeutic agents such as doxorubicin (DOX), paclitaxel, and cisplatin enhances tumor cytotoxicity via dual mechanisms: ROS-mediated oxidative stress from PDT and the cytotoxic effects of chemotherapeutics [23]. For instance, low-temperature-sensitive liposomes (LTSLs) encapsulating DOX and combined with multibranched gold nanoantennas have shown enhanced tumor accumulation and controlled drug release under NIR-induced hyperthermia (~42 °C) [147]. This synergistic approach resulted in superior tumor regression in TNBC models compared to either monotherapy, highlighting the potential of spatiotemporally controlled, stimulus-responsive liposomal delivery. Hydrogel-based PDT-chemo systems further exemplify the potential of combinational therapy. Ce6-DOX-MnO_2_-loaded calcium–alginate hydrogels can fragment into nanoparticles upon laser irradiation, penetrate deeper tumor regions, and generate oxygen in situ to overcome hypoxia [148,149]. Additionally, Mn^2+^ released from MnO_2_ activates the cGAS/STING pathway, augmenting immunogenic cell death and enhancing antitumor immune responses [150]. In vitro studies demonstrated over fivefold increased ROS production, and in vivo TNBC models exhibited significant tumor inhibition with minimal systemic toxicity. Multifunctional nanoplatforms based on metal–organic frameworks (MOFs) have been engineered to co-deliver chemotherapeutic drugs and photosensitizers, simultaneously improving drug delivery, ROS generation, and PDT efficacy [151]. Such platforms address PDT resistance caused by tumor metabolic reprogramming while enabling real-time imaging and treatment monitoring. In another work by Panikar et al. (2018) reports the first development of novel anti-HER2 peptide-conjugated ligand-targeted nanoliposomes (LTLs) designed for combined chemo-photodynamic therapy against HER2-positive breast cancer [17]. The LTL platform simultaneously encapsulates doxorubicin (DOX) for chemotherapy and methylene blue-conjugated upconversion nanoparticles (MB@UCNPs) for NIR-activated photodynamic therapy (PDT) and bioimaging, creating a true theranostic system. Target specificity was achieved through a novel anti-HER2 peptide screened from a phage display library, confirmed by selective uptake in HER2-positive SKBR-3 cells over HER2-negative MCF-7 cells. Upon 975 nm NIR laser excitation, energy transfer from UCNPs to MB generated cytotoxic ROS [134,135], enabling deep-tissue PDT activation. The combinational therapy achieved 95% reduction in cell viability, significantly outperforming chemotherapy alone (77%) and PDT alone (84%). Notably, methylene blue’s additional ability to downregulate P-glycoprotein addresses chemotherapy resistance, while liposomal encapsulation prevents MB accumulation in non-target cells, preserving PDT efficiency at the tumor site. Efficacy was further validated in 3D SKBR-3 tumor spheroids, where combinational therapy suppressed spheroid viability by 66%, with enhanced LTL penetration at the tumor margin improving cellular uptake. These results collectively position peptide-conjugated theranostic LTLs as a promising, clinically translatable platform for targeted, multimodal breast cancer management.

### 6.2. PDT Combined with Photothermal Therapy (PTT)

PDT combined with PTT leverages thermal and oxidative stresses to induce synergistic tumor cell death. Metallic nanostructures, such as gold nanoshells, nanorods, or multibranched nanoantennas, incorporated within liposomes, absorb NIR light to generate localized hyperthermia [152]. This not only ablates tumor tissue but also accelerates photosensitizer activation and drug release, enhancing PDT efficacy.

Tumor-responsive nanoassemblies, which remain inactive in systemic circulation and selectively disassemble upon exposure to extracellular matrix markers like Extra-domain B fibronectin (EDB-FN), can trigger synchronized PDT and PTT in the tumor microenvironment [153]. This selective activation mitigates systemic toxicity while enabling precise control over therapeutic action. PTT also enhances vascular permeability and tumor penetration, facilitating deeper delivery of photosensitizers and chemotherapeutic drugs. In the recent study by Zhang et al. (2026), the development of a multifunctional, tumor-targeted nanoparticle platform (MnO/Ce6@PDA@CCM) for synergistic breast cancer therapy [154]. The system consists of a polydopamine (PDA) core loaded with the photosensitizer chlorin e6 (Ce6) and coated with manganese oxide (MnO), followed by tumor cell membrane encapsulation to enable homotypic targeting and enhanced tumor accumulation. Upon dual near-infrared (660 and 808 nm) laser irradiation, the nanoparticles generate robust photothermal and photodynamic effects, producing high levels of reactive oxygen species (ROS) that induce mitochondrial and membrane damage in tumor cells. In the tumor microenvironment, MnO degradation releases Mn^2+^ ions, which enhance T1-weighted MRI contrast and promote chemodynamic therapy via Fenton-like reactions [154]. This light-responsive, imaging-guided nanoplatform offers a promising strategy for combinatorial photothermal, photodynamic, and chemodynamic therapy in breast cancer. Also, our group developed a multifunctional UCNPs-AuNPs nanohybrid for simultaneous breast cancer imaging and photothermal therapy. NaYF_4_:Yb, Er upconversion nanoparticles were covalently decorated with tailored 8 nm gold nanoparticles via a sub-1 nm linker in a rapid 10 min reaction [146]. Upon 975 nm near-infrared excitation, energy transfer from UCNPs to AuNPs generated localized hyperthermia, raising the temperature from 37 °C to ~42 °C within 5 min, sufficient to reduce MCF-7 breast cancer cell viability by over 60%, while remaining essentially non-toxic in the absence of NIR irradiation [146]. The same excitation simultaneously enabled ratiometric temperature sensing (25–50 °C) using the 525/545 nm green emission bands, allowing real-time monitoring and control of heat generation during therapy. The minimally quenched red emission (659 nm) was exploited independently for fluorescence imaging and cancer cell tracking [146]. This single nanoplatform therefore integrates photothermal therapy, nano-thermometry, and fluorescence imaging into one theranostic system, representing a promising approach for precision, image-guided breast cancer treatment.

In another recent study by Li et al. (2026), a pH-responsive, charge-reversal liposomal nanoplatform (IR-E@Lip) co-encapsulating Erianin and IR780 was developed to enhance multimodal breast cancer therapy [155]. The system maintains a negative surface charge during systemic circulation to prolong stability, but undergoes charge conversion in the acidic tumor microenvironment, promoting tumor-selective uptake and mitochondrial accumulation. Upon near-infrared irradiation, IR780 mediates photothermal and photodynamic effects, generating reactive oxygen species (ROS) and mild hyperthermia that disrupt mitochondrial integrity and induce immunogenic cell death (ICD) [155]. Concurrently, Erianin suppresses therapy-induced PD-L1 upregulation, counteracting immune evasion and amplifying antitumor immune responses. By integrating chemotherapy, photothermal therapy (PTT), and photodynamic therapy (PDT) within a single liposomal platform, this approach achieves potent tumor suppression with reduced systemic toxicity, offering a promising strategy for synergistic and immune-enhanced breast cancer treatment. Collectively, these studies underscore the power of integrating PDT and PTT within smart, tumor-responsive nanoplatforms to achieve synergistic cytotoxicity, enhanced tumor targeting, and immune activation. Such multifunctional strategies represent a promising direction for precision-guided and clinically translatable cancer therapy.

### 6.3. PDT Combined with Immunotherapy

Integrating PDT with immunotherapy allows PDT-induced immunogenic cell death (ICD) to synergize with immune checkpoint modulation, enhancing systemic anti-tumor immunity [156]. Liposomal prodrugs can be engineered to remain inactive during circulation and selectively release chemotherapeutics and photosensitizers in the acidic tumor microenvironment [23]. For example, caspase-3-responsive unimolecular prodrugs activate synchronously upon tumor-specific stimuli, promoting ICD, reversing immunosuppressive tumor conditions, and increasing CD8^+^ T cell infiltration. Such combinational approaches not only improve local tumor control but also help prevent metastasis and recurrence, addressing a major challenge in aggressive TNBC therapy [157]. In a recent study, Li et al. (2025) designed PDT biomimetic liposomes (PB Lipo), formulated from phospholipid compositions mimicking the native ER membrane, which is a notable strength of this work [143]. By exploiting membrane fusion as the primary mechanism of ER accumulation, the authors demonstrate a sophisticated approach to subcellular drug delivery that goes beyond passive tumor targeting. The incorporation of indocyanine green, an FDA-approved photosensitizer, further underscores the translational potential of this platform, offering a clinically viable pathway toward eventual application [143]. Upon NIR laser activation, the ER-localized PB Lipo induces profound ER stress, serving as a powerful trigger for ICD, a process that exposes damage-associated molecular patterns capable of priming an antitumor immune response. The downstream immunological cascade reported here, including enhanced dendritic cell maturation, robust CD4+ and CD8+ T cell activation, and elevated cytotoxic cytokine secretion, provides strong mechanistic support for the observed antitumor efficacy in xenograft TNBC models [143]. The combination with anti-PD-L1 antibody therapy represents a logical and well-justified extension of the platform, capitalizing on the immunostimulatory groundwork laid by PDT-induced ICD to maximize responsiveness to checkpoint blockade. Together, these findings position PB Lipo-mediated immuno-photodynamic therapy as a promising combinatorial strategy with the potential to meaningfully address the unmet clinical need in TNBC management.

In another work by Yang et al. (2024), using mitochondria-targeting Lipo-Ce6, the authors convincingly demonstrate that PDT-generated ROS triggers mitochondrial damage, mtDNA cytoplasmic release, and subsequent NLRP3/Caspase-1/GSDMD pathway activation, culminating in pyroptotic cell death [158]. The pharmacological inhibition experiments using NAC and ethidium bromide provide solid functional validation of this mechanistic model. A notable strength is the concurrent induction of immunogenic cell death alongside pyroptosis, evidenced by calreticulin exposure, HMGB1 and ATP release, effectively converting an immunologically cold tumor microenvironment into a more responsive one [158]. In vivo results further confirm robust tumor suppression, and the addition of checkpoint inhibitor BMS202 meaningfully amplifies immune cell infiltration and overall antitumor efficacy [158]. Safety assessments support the translational viability of this combined approach.

Overall, all this work presents a well-supported mechanistic framework and positions PDT combined with immune checkpoint blockade as a rational and promising therapeutic strategy for breast cancer, warranting further clinical exploration.

### 6.4. X-Ray Induced Photodynamic Therapy (X-PDT)

X-ray-induced Photodynamic Therapy (X-PDT) has emerged as a highly promising strategy to overcome one of the major limitations of conventional photodynamic therapy (PDT), namely the poor penetration depth of visible and near-infrared light in deep-seated tumors [159,160]. By utilizing X-rays as the excitation source, X-PDT enables noninvasive activation of photosensitizers within deep tumor tissues, resulting in the generation of reactive oxygen species (ROS) that induce tumor cell death through apoptosis, necrosis, mitochondrial dysfunction, and oxidative membrane damage [161]. This approach is particularly attractive for difficult-to-treat malignancies such as Triple-Negative Breast Cancer (TNBC), where conventional therapies often fail because of tumor aggressiveness, metastasis, therapeutic resistance, and severe treatment-associated toxicity [57].

Recent advances in nanotechnology have significantly improved the therapeutic efficacy of X-PDT through the development of multifunctional nanocarriers capable of enhancing ROS production, improving tumor targeting, and overcoming the hypoxic tumor microenvironment. One such strategy involved the development of liposomal nanocarriers co-loaded with protoporphyrin IX (PPIX) and the oxygen carrier perfluorooctyl bromide (PFOB). Since oxygen availability is critical for ROS generation during PDT, the incorporation of PFOB created an oxygen-enriched tumor microenvironment that substantially enhanced X-ray-triggered ROS production from PPIX under hypoxic conditions [57]. Under low-dose X-ray irradiation (2 Gy), a 4.9-fold increase in intracellular ROS generation and significantly increased tumor cell death compared with untreated controls, highlighting the importance of oxygen-supplemented nanosystems in improving X-PDT efficacy in hypoxic tumors [57].

In parallel, other studies have explored X-ray-responsive nanoparticles capable of directly generating cytotoxic singlet oxygen upon irradiation. Copper-cysteamine (Cu-Cy) nanoparticles demonstrated strong therapeutic potential as X-ray-activatable photosensitizers for deep tumor therapy [162]. Upon X-ray exposure, Cu-Cy nanoparticles generated singlet oxygen through energy-transfer mechanisms similar to conventional PDT, leading to oxidative cellular damage. Furthermore, conjugation of Cu-Cy nanoparticles with pH-low insertion peptide (pHLIP) improved tumor targeting and enhanced radiation-mediated tumor reduction in mouse models, suggesting that targeted X-ray-responsive nanoplatforms can further improve antitumor efficacy while maintaining the deep tissue penetration advantages of X-rays [162].

**Table 2:** Comparative overview of liposomal nanoplatforms for photodynamic therapy (PDT) in breast cancer, including physicochemical properties, photosensitizers, irradiation conditions, combinational therapeutic strategies, breast cancer models, and tumor subtypes.

Liposome Size (nm)	Photosensitizer	Irradiation Wavelength (nm) or X-Ray Dose (Gy)	Combinational Strategy	Murine Breast Cancer Model	Human Breast Cancer Model	Breast Cancer Type	Reference
200	ICG	810 nm	Chemotherapy: Paclitaxel	N/A	KPL-1 cells	ER+/HER2-	[163]
246.6 ± 1.8 nm	protoporphyrin IX (PPIX)	2 Gy	X-ray-induced photodynamic therapy (X-PDT): Uses perfluorooctyl bromide (PFOB) to treat hypoxia	N/A	Hs578	TNBC	[57]
208.3 ± 1.07 nm	ICG	810 nm	Cuproptosis inducer elesclomol-Cu (ES-Cu)	N/A	MCF-7	ER+/PR+/HER2-	[74]
81.5 ± 4.2 nm	IR820	808 nm	Loaded with BMS-202, a small molecule PD-L1 inhibitor and chemotherapy drug tirapazamine	N/A	4T1	TNBC-like: ER-/PR-/HER2-	[164]
90 nm	Pyropheophorbide-a (PPa)	660 nm	Redox-active PDT and PDT-triggered release of indoleamine 2,3-dioxygenase inhibitor for enhanced anti-tumor immune response	N/A	4T1	TNBC-like: ER-/PR-/HER2-	[165]
122.4 nm	MBDP: Lysosome-targeted NIR BODIPY photosensitizer,	650 nm	Catalase to enhance PDT and doxorubicin as a chemotherapy drug	N/A	4T1	TNBC-like: ER-/PR-/HER2-	[166]
					MCF-7	ER+/PR+/HER2-	
90 nm	Methylene blue	975 nm	N/A	N/A	SKBR3	ER-/PR-/HER2+	[18]
					MCF-7	ER+/PR+/HER2-	
90 nm	Methylene blue	975 nm	Combined with upconversion nanoparticles for bioimaging and emission-based photoactivation, and doxorubicin as a chemotherapy drug	N/A	SKBR3	ER-/PR-/HER2+	[17]
					MCF-7	ER+/PR+/HER2-	
160 nm	Methylene blue	660 nm	N/A	N/A	4T1	TNBC-like: ER-/PR-/HER2-	[167]
95 nm	hexadecylamine-conjugated chlorin e6	660 nm	Theranostic combination of ^64^Cu-based Positron emission imaging, PDT and hypoxia-mediated release of AQ4N (a non-toxic prodrug)	N/A	4T1	TNBC-like: ER-/PR-/HER2-	[168]
~228 nm	Ce6	660 nm	Ce6-mediated PDT with αPD-L1 immunotherapy	N/A	4T1	TNBC-like: ER-/PR-/HER2-	[169]
150 nm	Aluminum phtalocynanine chloride (AlCIPc)	660 nm	N/A	N/A	MCF-7	ER+/PR+/HER2-	[170]
					MDA-MB-231	(TNBC)	

More recently, purely organic phosphorescent nanoscintillators have emerged as an alternative strategy for X-PDT, avoiding the potential toxicity concerns associated with heavy-metal-containing sensitizers. An organic nanoscintillator based on 9,9′-(6-iodophenoxy-1,3,5-triazine-2,4-diyl)bis(9H-carbazole) (ITC) was engineered to enhance intersystem crossing and triplet exciton generation through the incorporation of iodine, oxygen, and nitrogen atoms [161]. Water-soluble ITC nanoparticles (ITC-NPs), synthesized using PEG-b-PPG-b-PEG (F127), exhibited stable spherical morphology and efficient X-ray-triggered singlet oxygen production. Mechanistic studies in 4T1 breast cancer cells revealed robust intracellular singlet oxygen generation, increased lipid peroxidation, mitochondrial membrane depolarization, DNA damage, and suppression of colony formation following X-PDT treatment. Importantly, the therapeutic effect was primarily attributed to photodynamic ROS generation rather than radiosensitization alone [161].

Notably, ITC-NPs demonstrated excellent in vivo biosafety, with no significant hematological, hepatic, renal, or cardiac toxicity following repeated administration. In tumor-bearing mouse models, the nanoparticles showed efficient tumor accumulation and prolonged circulation, resulting in significant tumor suppression under an extremely low X-ray dose of only 0.4 Gy, outperforming radiotherapy alone [161]. Histological analysis further confirmed extensive tumor apoptosis and structural destruction in treated tumors. Collectively, these studies demonstrate that nanotechnology-assisted X-PDT, including oxygen-supplemented liposomes [57], targeted Cu-Cy nanoparticles [162], and organic nanoscintillators [161], represents a powerful and minimally invasive therapeutic platform for deep and hypoxic cancers, with substantial potential for future clinical translation.

## 7. Clinical Investigation and Translational Perspectives of PDT in Breast Cancer

Photodynamic therapy (PDT) has established a clinically relevant role in breast cancer management, particularly for patients with recurrent, locally advanced, or metastatic disease who have exhausted standard therapeutic options. Its primary advantage lies in spatially controlled tumor ablation with reduced systemic toxicity compared with chemotherapy and radiotherapy. However, despite strong mechanistic rationale and early clinical promise, translation into routine oncological practice has progressed more slowly than anticipated due to persistent biological and technological constraints.

Early-phase clinical studies provide encouraging proof-of-concept evidence. In a Phase I/IIa trial (NCT02872064), intravenous verteporfin followed by interstitial laser irradiation induced measurable tumor necrosis in 11 of 12 patients with early-stage invasive ductal carcinoma, demonstrating high spatial selectivity of PDT-mediated cytotoxicity. In metastatic breast cancer, Morrison et al. reported variable responses ranging from partial coagulation to complete regression in cutaneous lesions treated with Photofrin-based PDT. Similarly, Zhang et al. demonstrated that combining PDT with radiotherapy nearly doubled complete response rates compared with radiotherapy alone, while also accelerating response kinetics [171]. Laser immunotherapy approaches using indocyanine green and glycated chitosan have been further reported to provide clinical benefit in approximately 75% of late-stage patients. Ongoing Phase II clinical trials (NCT02939274, NCT05374915) are expected to provide more rigorous validation of efficacy, safety, and patient stratification strategies, which are essential for defining the clinical positioning of PDT. Despite these encouraging outcomes, clinical response remains highly heterogeneous. A central limitation is the suboptimal performance of conventional photosensitizers, which often exhibit poor tumor selectivity, aggregation in biological fluids, and reduced reactive oxygen species (ROS) generation. In addition, the oxygen-dependent nature of PDT significantly limits efficacy in hypoxic tumor regions, which are prevalent in aggressive breast cancers. Although aggregation-induced emission photosensitizers and oxygen-replenishing nanoplatforms have been developed to mitigate these challenges, their clinical validation remains limited.

Another major barrier is the disconnect between preclinical models and human disease complexity. Standard murine tumor models fail to fully replicate the vascular heterogeneity, stromal density, and immune microenvironment of human breast cancer, resulting in an overestimation of therapeutic efficacy [172]. While patient-derived xenografts and three-dimensional organoid systems provide improved physiological relevance, their adoption remains limited due to cost, technical complexity, and lack of standardization [172,173]. Manufacturing and scalability further constrain clinical translation. Liposomal and nanocarrier-based photosensitizer systems often undergo physicochemical alterations during scale-up, affecting stability, biodistribution, and targeting efficiency. Moreover, surface functionalization with antibodies or peptides introduces additional complexity, significantly increasing production costs while requiring strict adherence to Good Manufacturing Practice (GMP) and regulatory frameworks established by agencies such as the FDA and EMA [174,175,176]. Although emerging techniques such as microfluidic synthesis and continuous flow production have improved reproducibility, scalable manufacturing of clinically compliant nanomedicines remains a major unresolved challenge. From a translational perspective, the field is increasingly shifting toward integrated multimodal therapeutic strategies rather than standalone PDT. Combinational approaches incorporating chemotherapy, photothermal therapy, immunotherapy, and X-ray–activated PDT have demonstrated synergistic effects by simultaneously addressing hypoxia, limited drug penetration, and immune suppression. These strategies highlight the growing importance of system-level design in overcoming intrinsic limitations of single-modality PDT.

Looking forward, the clinical translation of PDT is expected to be driven by the convergence of personalized nanomedicine, computational optimization, and clinically integrated delivery systems. Personalized nanomedicine enables tailoring of liposomal photosensitizer platforms according to patient-specific tumor characteristics [177], including receptor expression profiles, immune microenvironment composition, and hypoxic status, thereby improving therapeutic predictability in heterogeneous breast cancer subtypes. In parallel, artificial intelligence (AI)–assisted design and optimization approaches are emerging as powerful tools to predict nanocarrier performance [178,179], optimize formulation parameters, and stratify patients based on predicted response, although current applications remain largely exploratory. Another key direction is image-guided theranostics, in which diagnostic imaging modalities such as fluorescence imaging, magnetic resonance imaging (MRI), or positron emission tomography (PET) are integrated with PDT platforms to enable real-time monitoring of biodistribution, treatment response, and adaptive therapy modulation [168]. This capability is particularly valuable in breast cancer, where spatial heterogeneity in oxygenation and drug delivery significantly affects therapeutic outcomes.

From a manufacturing standpoint, scalable and reproducible GMP-compliant production of liposomal nanocarriers remains essential for clinical translation. Advances in microfluidic-assisted synthesis and continuous manufacturing platforms offer promising routes to improve batch consistency and reproducibility, although maintaining physicochemical stability during scale-up continues to present challenges. In parallel, regulatory considerations, including compliance with FDA and EMA requirements for nanomedicine characterization, toxicity evaluation, and long-term safety profiling, will play a decisive role in determining clinical approval pathways [180]. Addressing these regulatory, manufacturing, and biological challenges in a coordinated manner will be critical for successful clinical translation.

PDT for breast cancer demonstrates a strong mechanistic rationale and encouraging early clinical outcomes, and liposomal nanocarrier systems have significantly expanded its therapeutic potential. However, a substantial translational gap remains between preclinical success and routine clinical implementation. Bridging this gap will require advances in next-generation photosensitizers, more predictive preclinical models, scalable and regulatory-compliant manufacturing strategies, and rigorously designed clinical trials with appropriate patient stratification. Collectively, these developments will determine whether PDT evolves from an adjunct modality into a clinically established component of precision breast cancer therapy.

## 8. Discussion: Challenges and Future Perspectives

Photodynamic therapy (PDT) represents a promising minimally invasive strategy for breast cancer treatment, offering spatially controlled tumor ablation with reduced systemic toxicity compared with conventional chemotherapy and radiotherapy. However, its clinical translation remains constrained by fundamental biological barriers, including tumor hypoxia, limited light penetration in deep-seated lesions, and inefficient intracellular delivery of photosensitizers. Collectively, these limitations significantly reduce reactive oxygen species (ROS) generation and contribute to heterogeneous therapeutic outcomes in breast cancer patients. To address these challenges, nanocarrier-based delivery systems, particularly liposomal platforms, have emerged as one of the most advanced and clinically relevant strategies. Liposomes improve photosensitizer solubility and stability, enhance tumor accumulation via passive and active targeting mechanisms, and enable controlled intracellular delivery. Importantly, they also facilitate multimodal therapeutic integration, allowing PDT to be combined with chemotherapy, photothermal therapy, gene therapy, immunotherapy, and X-ray–induced PDT (X-PDT). Among these, combinational PDT strategies have consistently demonstrated superior therapeutic efficacy compared with monotherapy by simultaneously addressing tumor hypoxia, drug resistance, and immune evasion. A key strength of liposomal PDT systems lies in their ability to function as multifunctional theranostic platforms, integrating diagnostic imaging with therapeutic delivery. This enables real-time monitoring of biodistribution and treatment response, which is particularly relevant for heterogeneous and treatment-resistant breast cancer subtypes. However, despite these advantages, the relative performance of different nanoplatforms remains difficult to directly compare due to variability in photosensitizer type, tumor models, irradiation parameters, and formulation characteristics, highlighting the need for standardized evaluation frameworks.

Importantly, our group [17,18,134,135,146] and others have significantly contributed to advancing PDT-based combinational nanotherapeutic strategies for breast cancer. These studies have focused on rational nanostructure design to enhance ROS production, improve tumor selectivity, and overcome hypoxic and deep-tissue delivery limitations. Collectively, these advances demonstrate that rationally engineered liposomal systems can substantially improve PDT efficacy, particularly in resistant breast cancer phenotypes, while reducing systemic toxicity. Despite these significant advances, several translational barriers remain unresolved. Large-scale and reproducible manufacturing of liposomal nanocarriers remains challenging due to batch-to-batch variability and sensitivity of physicochemical properties during scale-up. In addition, regulatory compliance under Good Manufacturing Practice (GMP) frameworks imposes substantial constraints on surface functionalization strategies and clinical-grade reproducibility. Biological heterogeneity of breast tumors, including variability in vascularization, stromal density, and immune microenvironment composition, further complicates clinical translation and contributes to inconsistent therapeutic outcomes. Therefore, future progress will depend on the development of standardized, scalable, and clinically adaptable nanoplatforms, alongside improved preclinical models that better recapitulate human tumor complexity. Optimization of nanocarrier design to enhance tumor specificity, intracellular delivery efficiency, and pharmacokinetic stability will be essential for improving clinical predictability. In parallel, integration of PDT with complementary therapeutic modalities and real-time imaging systems is expected to further enhance precision and therapeutic control.

In summary, liposome-enabled PDT and combinational nanotherapies provide a powerful and versatile platform for overcoming the intrinsic limitations of conventional breast cancer therapies. However, bridging the gap between preclinical success and clinical translation will require coordinated advances in nanomaterial engineering, biological validation, scalable manufacturing, and regulatory alignment. Continued interdisciplinary development in these areas will be critical for transforming PDT-based strategies into clinically effective and personalized breast cancer therapies.

## Data Availability

No data was added.
